# Study of the HIV-2 Env Cytoplasmic Tail Variability and Its Impact on Tat, Rev and Nef

**DOI:** 10.1371/journal.pone.0079129

**Published:** 2013-11-01

**Authors:** Nordine Bakouche, Anne-Thérèse Vandenbroucke, Patrick Goubau, Jean Ruelle

**Affiliations:** Institut de recherche expérimentale et clinique, Université catholique de Louvain, Brussels, Belgium; Istituto Superiore di Sanità, Italy

## Abstract

**Background:**

The HIV-2 env’s 3’ end encodes the cytoplasmic tail (CT) of the Env protein. This genomic region also encodes the rev, Tat and Nef protein in overlapping reading frames. We studied the variability in the CT coding region in 46 clinical specimens and in 2 reference strains by sequencing and by culturing. The aims were to analyse the variability of Env CT and the evolution of proteins expressed from overlapping coding sequences.

**Results:**

A 70% reduction of the length of the CT region affected the HIV-2 ROD and EHO strains *in* *vitro* due to a premature stop codon in the *env* gene. In clinical samples this wasn’t observed, but the CT length varied due to insertions and deletions. We noted 3 conserved and 3 variable regions in the CT. The conserved regions were those containing residues involved in Env endocytosis, the potential HIV-2 CT region implicated in the NF-kB activation and the potential end of the lentiviral lytic peptide one. The variable regions were the potential HIV-2 Kennedy region, the potential lentiviral lytic peptide two and the beginning of the potential lentiviral lytic peptide one. A very hydrophobic region was coded downstream of the premature stop codon observed *in* *vitro*, suggesting a membrane spanning region. Interestingly, the nucleotides that are responsible for the variability of the CT don’t impact rev and Nef. However, in the Kennedy-like coding region variability resulted only from nucleotide changes that impacted Env and Tat together.

**Conclusion:**

The HIV-2 Env, Tat and Rev C-terminal part are subject to major length variations in both clinical samples and cultured strains. The HIV-2 Env CT contains variable and conserved regions. These regions don’t affect the rev and Nef amino acids composition which evolves independently. In contrast, Tat co-evolves with the Env CT.

## Introduction

The human immunodeficiency virus type 2 (HIV-2) *env* gene encodes the envelope polyglycoprotein (Env) that is cleaved inside the cell by an endogenous protease and leads to the production of two glycoproteins (gpSU and gpTM) [1,2]. 

gpSU is present at the surface of the envelope while gpTM is a transmembrane glycoprotein. The gpTM contains four major parts: the fusion peptide and the heptad repeats which are located outside the virus [3-5], the transmembrane region [6], and the C-terminal domain which is the only internal region of Env and is called the cytoplasmic tail (CT). 

Little data is known about the HIV-2 CT, but the HIV-1 CT contains subregions namely, from its N-terminal to C-terminal part, the endocytosis signal sequence, the Kennedy sequence, three lentiviral lytic peptides (LLP) and a final di-Leucine motif [7-18]. The latter is also involved in the process of Env endocytosis [10,11]. The Kennedy sequence contains epitopes that are recognised by antibodies when they are expressed in rabbits [12-14]. Finally, the three HIV-1 LLPs are regions that can alter the permeability of the cell membrane [15-18]. Except for the identification of the endocytosis signal, no systematic comparison of the patterns of the HIV-1 and HIV-2 CT has been published to date [19]. 

The HIV-2 *env* gene contains the nucleotide sequences that encode Tat, Rev and the N-terminal part of Nef in overlapping reading frames. The 3’end of the *env* gene that expresses HIV-2 CT is the region where the overlap is the most important as 4 proteins are expressed from that sequence. The study of the 3’ end of the *env* gene constitutes an interesting model for the characterisation of the poorly known HIV-2 CT and for a study of the evolution of proteins expressed from different reading frames in a single sequence. 

For this purpose, we sequenced the CT coding region from *in vitro* adapted strains and from clinical samples at different stages of the disease. The *env* coding sequences obtained were then used to analyse the CT variability, and to study the impact of the CT variability on the other proteins expressed from the same nucleotides sequence. 

## Results

### HIV-2 Env CT full-length is not mandatory *in vitro*

We sequenced the reference strains ROD and EHO (representing respectively HIV-2 groups A and B) after several passages *in vitro* in H9 cells. We found several differences when we compared the sequences of our cultured virus with the published reference sequences: ROD (Genbank:M15390) and EHO (Genbank:U27200), (Table 1). The most important difference was the replacement of a tryptophan codon (TGG) by a stop codon at the 748^th^ EHO *env* codon and 750^th^ ROD *env* codon (always TAG). We also confirmed this adaption of the CT length with three independent experiments in which the infectious clone pKP59-ROD, initially cloned from a clinical sample [20], was transfected in 293 cells and passaged several times on MT2, MT4 and H9 cells. This phenomenon was thus constant in various lymphoid lineages and was not strain-specific. 

**Table 1:** HIV-2 ROD and EHO reference strain aa sequence alteration after H9 cell line passages.

**HIV-2 ROD (group A)**			**HIV-2 EHO (group B)**		
Env Aa position	Aa in the reference	Aa in the *in vitro* selected virus	Env Aa position	Aa in the reference	Aa in the *in vitro* selected virus
727	R	R/Q	700	R	R/Q
728	G	E	720	P	P/L
749	P	L	723	K	K/E
750	W	STOP	724	D	D/E
792	R	R/K	725	R	R/W
793	D	D/N	739	N	N/D
794	W	W/STOP	740	N	N/D
796	R	R/K	741	R	R/G
798	R	R/K	747	P	P/L
800	A	A/T	748	W	STOP
815	A	T	756	P	L
821	R	R/K	757	I	I/F
840	R	K	761	R	R/K
841	G	G/E	788	P	P/L
848	R	R/K	793	P	L

In the table, we can observe: the Env position of the substitution, the aa substituted and the new aa (X/Y corresponding to a mixture of aa). CT starts at 701^st^ Env position for ROD and 699^th^ for EHO.

### The entire HIV-2 CT length is required *in vivo*

We sequenced the 46 CT coding region obtained from 27 different patients at various clinical stages (Table 2). Thirty of those 46 sequences were obtained from plasma RNA and 16 from proviral DNA samples. Although we observed that the CT length varied from 148 to 165 amino acids (aa) due to insertions and deletions in the DNA/RNA sequences, we did not find any premature stop codon. In contrast to what was observed *in vitro*, a full-length CT was found in all clinical samples. 

**Table 2:** Patient cohort description (N=27).

Genbank accession number	Plasma viral load (copies/mL)	CD4 count (cell/mL)	AIDS (CDC definition)	ARV before sampling	Gender	Age at sampling
KC748535	6450	166	Y	Y	M	56
KC748536	187000	227	N	Y	F	54
KC748538	3155	310	N	Y	M	53
KC748540	1420	330	N	Y	M	53
KC748544	1495	500	N	Y	M	48
KC748547	575	775	N	N	M	48
KC748548	565	287	N	N	M	44
KC748549	1065	434	N	N	M	44
KC748550	35750	266	N	Y	M	46
KC748551	2025	233	N	N	M	43
KC748552	1150	451	N	N	F	43
KC748553	555	219	N	N	M	41
KC748555	47435	N.A.	N	N	F	40
KC748556	405	581	Y	N	F	39
KC748557	1770	718	N	N	F	34
KC748558	435	N.A.	N	N	F	32
KC748559	480	N.A.	N	N	M	32
KC748561	4165	N.A.	N	N	F	28
KC748562	735	N.A.	N	N	M	21
KC748563	14800	N.A.	N	Y	M	10
KC748564	515000	100	Y	Y	M	33
KC748566	185	704	N	N	M	51
KC748567	5250	162	N	N	F	50
KC748577	<50	531	N	Y	F	47
KC748578	<50	492	Y	Y	F	42
KC748579	74	N.A.	N	N	F	35
KC748580	<50	356	Y	Y	M	50

**Figure 1:**
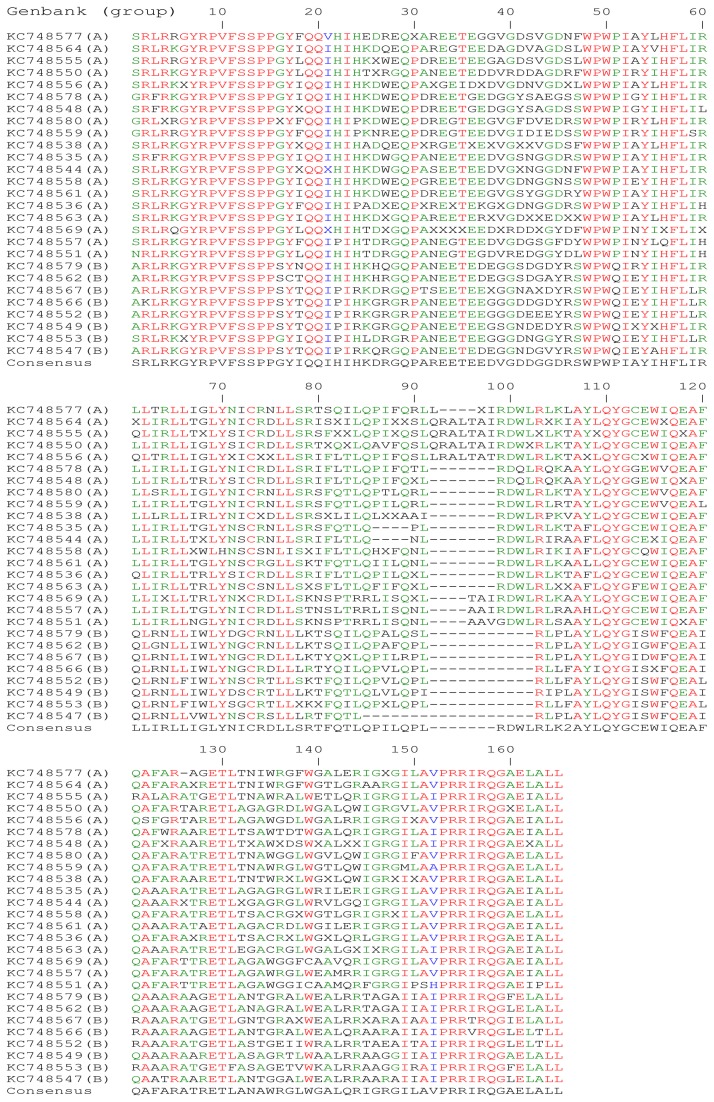
Alignment of 27 virus/provirus CT HIV-2 aa sequences (1 sequence per patient), classified by similarity. The GenBank accession number is followed by the HIV-2 group (A or B). To construct the alignment, only the most recent sample was included. The conserved aa are highlighted in red (>85% of conservation). In green appears the most represented aa for each position (>50% of conservation). Blue corresponds to variable positions with similar aa.

For each patient we show: accession number of the sequences identical to Figure 1, viral load, CD4 count, presence of AIDS (Yes=Y, No=N), antiretroviral (ARV) treatment before sampling, gender (Male=M, Female= F) and age at sampling. N.A.= Not available.

### Analysis of the sequences

The region studied by sequencing started from the *env* codon 701 for HIV-2 ROD and from *env* codon 698 for EHO up to the *env* stop codon. This region is homologous to the CT coding region of HIV-1. Figure 1 shows the alignment of 27 aa sequences including one sequence per patient. That alignment was used to study the Shannon Entropy (SE) of each aa position as well as the frequency of positions with a mixtures of aa along the CT region (Table 3). The SE can be seen as a measure of the variability of each position in the sequences [21]. The 165 positions of the alignment were divided into 7 regions, named A to G in Table 3. The CT started either with a serine, a glycine or an alanine. In all HIV-2 group A sequences a serine start was predominant and an alanine start was dominant in all HIV-2 group B sequences. All of our sequences ended with a leucine.

**Table 3:** Study of the variability and hydrophobicity of the different CT regions of HIV-2.

Region (CT aa positions)	Average SE	Average mixture of aa per position	Kyte-Doolittle scale score average	Average variation per position per alignment**	Average SE provius alignment/ virus alignment*
CT (1 to 165)	0.618	0.570	-0.295	0.511	0.229/0.200
A (0 to 20)	0.253	0.15	-0.616	0.250	0.121/0.068
B (21 to 47)	0.898	1.11	-1.813	0.889	0.360/0.283
B group A*	0.278	ND	ND	ND	ND
B group B*	0.193	ND	ND	ND	ND
C (48 to 68)	0.504	0.428	1.190	0.333	0.192/0.152
D (69 to 91)	0.807	0.957	0.023	0.479	0.232/0.252
E (92 to 125)	0.565	0.324	-0.195	0.441	0.189/0.208
F (126 to 136)	0.691	0.364	-0.495	0.272	0.292/0.216
G (137 to 165)	0.592	0.517	-0.032	0.724	0.228/0.191
G start (137 to 150)	0.968	1.071	0.216	1.286	0.360/0.312
G end (151 to 165)	0.242	0.067	-0.382	0.200	0.105/0.079

A: the first conserved region containing the endocytosis signal. B: the region containing the Kennedy-like sequences. C: the hydrophobic region D: LLP-2 domain. E: LLP-3 domain. F: LLP-3 / LLP-1 inter-region. G: LLP-1 region. ND: not determined. * Group A, B, provirus, virus alignement SE were normalized with the natural logarithm of the number of sequences. ** Results of the intra-patient study.

The first 20 CT aa positions were highly conserved (consensus SRLRKGYRPVFSSPPGYIQQ; region A). In this region, the conserved motif GYRPV corresponded to the endocytosis signal (position 6 to 10). A second well-conserved sequence extended from positions 13 to 20, with a consensus SSPPG/SYXQQ, where the X was a variable aa, G being present for all group A sequences and S for all group B sequences. The latter was homologous to a motif that was recently described and which interacts with NF-kB expression in SIVmac [22].

The most variable region of the full sequence extended from residues 21 to 47 with a consensus IHIHKDRGQPAREETEEDVGDDGGDRS in region B shown in Table 3. This region contains also the highest mixture of aa (Table 3). As this region contained aa homologous to the HIV-1 Kennedy sequence, we named it the Kennedy-like region. 

The 22 aa that follow this variable region (position 48 to 69, consensus WPWPIAYIHFLIR*LL*IR*LL*IGL; region B) were much more conserved, especially the two double leucine motifs coloured in italic (region C, Table 3). This region is also the most hydrophobic of the entire CT as shown by the Kyte-Doolittle scale score average (Table 3). The sequence of this CT region was interrupted after the first proline in the cultured virus, where a stop codon replaced the codon of the second tryptophan. 

The last 96 CT positions contained residues homologous to the three HIV-1 LLPs. The first part should contain a region homologous to the LLP-2 (position 70 to 91 consensus *Y*NICRD*LL*SRTFQTLQPILQPL; region D). This region was not conserved among our sequences and between the HIV-2 consensus and HIV-1 sequences, except for a punctual di-leucine motif and a tyrosine (in italic). Moreover, this region contained mixture of aa and we noted that some viruses had a deletion at the last position of this region (Figure 1).

The next region, homologous to the HIV-1 LLP-3 region, spanned from positions 92 to 125 (consensus -------RDWLRLKXAYLQYGCEWIQEAFQAFA, with a dash corresponding to the absence of aa as consensus; region E). The putative LLP-3 started by a complex situation with some sequences containing insertions or deletions in comparison with the consensus. Despite this complex situation, the rest of the sequences were not variable. 

**Figure 2:**
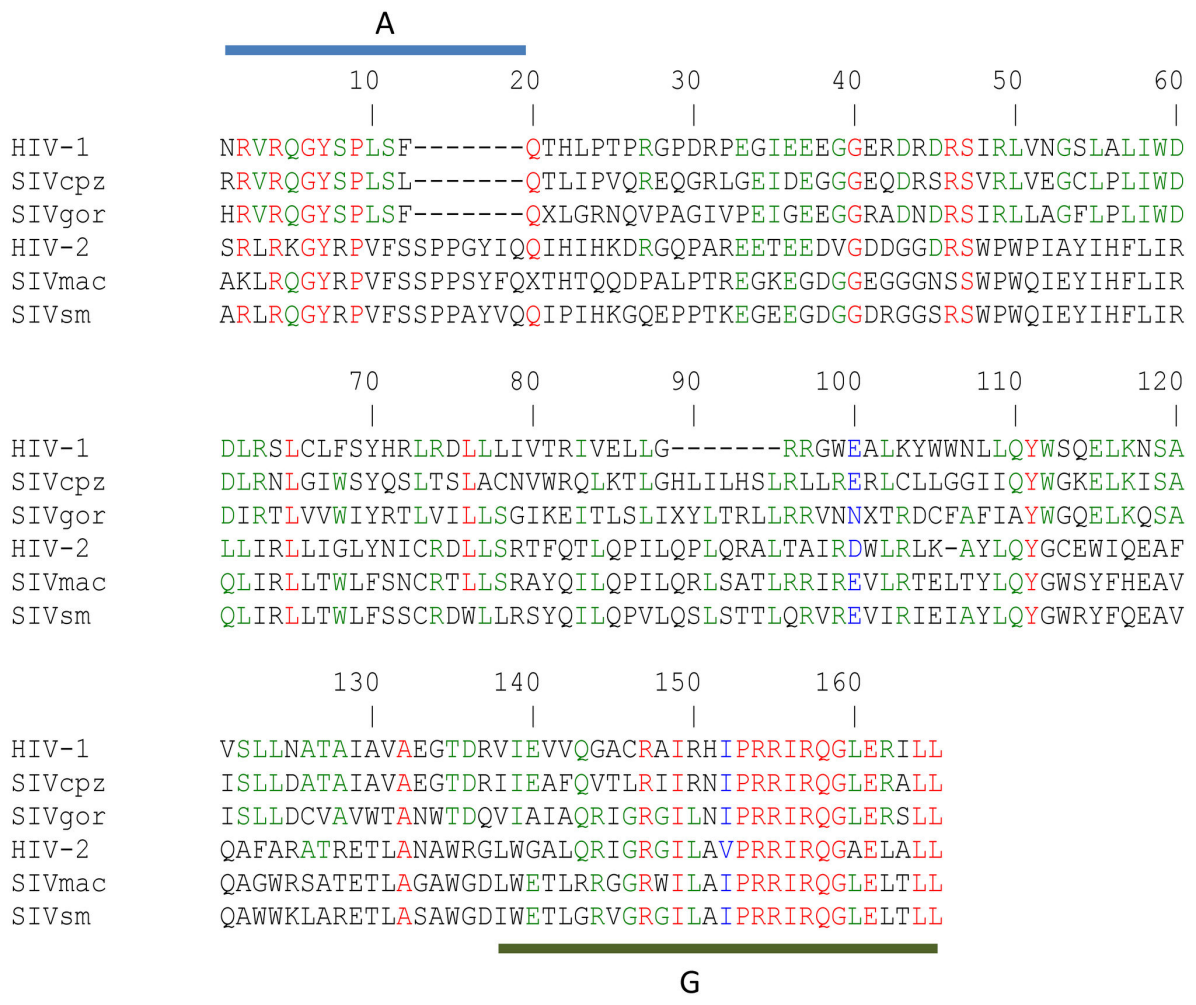
Env CT sequence alignment of the major primate lentiviruses. HIV-1: (Swiss-prot: P04578), SIVcpz: (Swiss-prot: P17281), SIVgor: (GenBank:FJ424865.1), HIV-2: CT consensus sequence from the article, SIVmac: (Swiss-prot: P08810) and SIVsm: (Swiss-prot: P12492). The conserved aa (in red) are aa of the endocytosis signal (region A) and the aa at the end of the LLP-1 region (region G). The aa implicated in SIVmac NF-kB activation (end of region A) are absent in HIV-1 and its lineage (SIVcpz and gor).

We divided the two final regions of our sequences in two arginine rich regions. The first region was a small LLP-3/LLP-1 inter-region (position 126 to 136 consensus RATRETLANAWR; region F, Table 3), and the second was the LLP-1 homologous sequence (position 137 to 165 consensuses GLWGALQRIGRGIL/AVPRRIRQGAELALL; with the final di-leucine motif; region G). The beginning of the LLP-1 region was highly variable (position 137 to 150, aa before the slash; G start, Table 3). This part contained also a lot of positions with mixtures of aa. In contrast, the end of the LLP-1 (aa after the slash) was the most constant part of the CT (G end, Table 3). Interestingly, this LLP-1 region was very rich in arginine and ended in all our sequences with the final di-leucine motif. Furthermore, the LLP-1 conserved part is also conserved among all primate lentiviruses (Figure 2). 

### HIV-2 CT sequences variability over time in infected individuals

The sequence evolution over time was studied in eight patients from whom we had more than one HIV-2 CT sequence available, which could be either exclusively viral or exclusively proviral. Nine alignments were performed with 25 sequences (1 alignment per patient except 1 patient with two alignments). As the number of sequences per alignment was limited, the use of the SE for the analysis of the variability was not suitable. Therefore, we measured the number of times where a CT position varied in each of the intra-patient alignments (Table 3). We observed that 40% of the positions varied over time in contrast to 76% of variable positions in the inter-patient alignment. The variable and constant regions were similar between the intra-patient and the inter-patient study. However, the regions D and F were less variable in the intra-patient study compared to the inter-patient evaluation (Table 3).

### Provirus-virus sequences comparison

We compared provirus and virus sequences from 4 patients for whom the sequences for both the provirus and the virus were available. We observed similar differences compared to the previous intra-patient study virus, but in one case a small insertion was present in the provirus and absent from the plasma RNA. Notably, this insertion was located in the LLP-3 complex coding region: virus (Genbank: KC748547) and provirus (Genbank: KC748572). As the number of patients was not sufficient to conclude that CT variability from RNA virus samples and DNA provirus is similar, we made two alignments with only one provirus or one virus sample per patient, resulting in 10 proviral DNA sequences (6 from group A) and 21 viral sequences (15 from group A), respectively. We measured the normalised SE from those alignments, by the natural log of the number of sequences (Table 3). The mean of the normalised SE was slightly more important in proviral DNA, possibly due to different ratios in group A and B sequences. However, the variable and constant regions were similar between provirus and virus sequence alignments. Therefore the choice of provirus or virus sequences did not affect the definition of variable and constant regions. 

### Influence of Tat, Rev and Nef on HIV-2 CT variability

The sequences of the *env* gene that we analysed are shared with the 2^nd^ exon of *tat* and *rev* and with the first half of *nef*. We analysed the reciprocal impact of Tat, Rev and Nef variability on the HIV-2 CT regions. 

The 2^nd^ exon of *tat* Open Reading Frame (ORF) starts at the 16^th^ /17^th^ codon of the CT, with the first nucleotides of each *tat* codons corresponding to the 2^nd^ nucleotide of each *env* codons. The beginning of the 2^nd^ *tat* exon is followed, 5 *env* codons later, by the beginning of the 2^nd^ exon of *rev*, with the first nucleotides of each *rev* codons corresponding to the 3^rd^ nucleotide of each *env* codon. Nef starts to be expressed at the 110^th^ /111^th^ CT codon, and is translated in the same reading frame as *tat*. 

**Figure 3:**
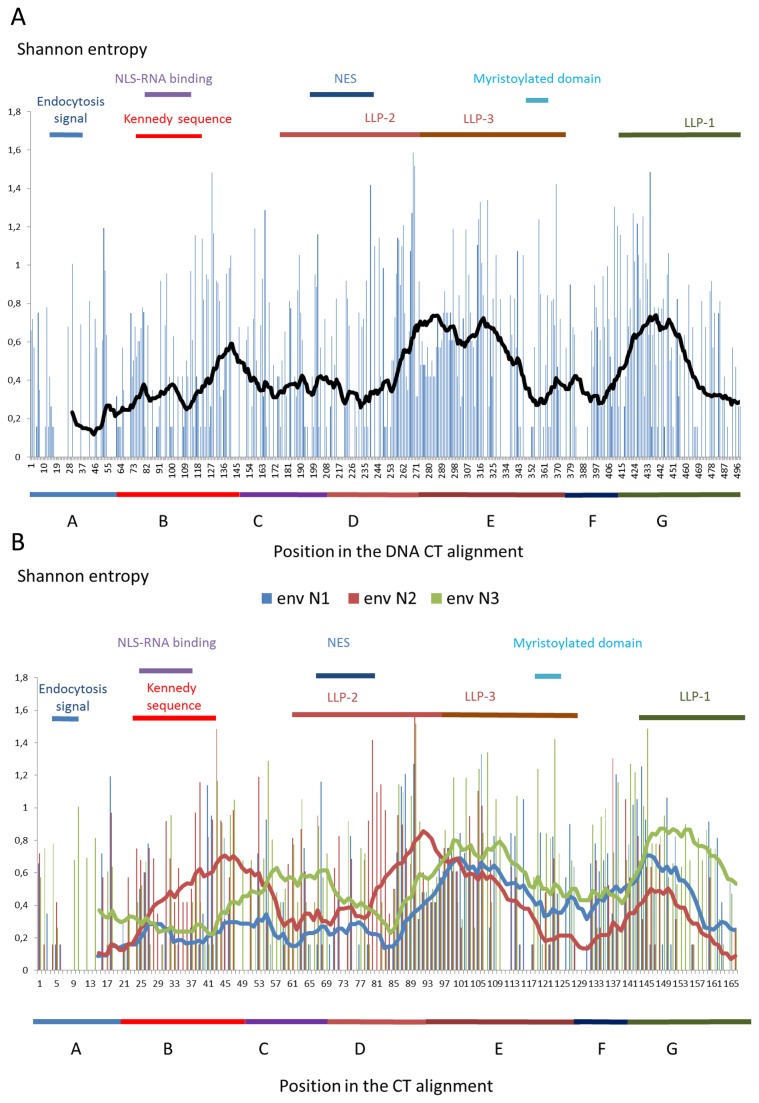
SE plots of the CT coding nucleotides. We created a SE plot for all the nucleotides (A) or for each nucleotide by codon (B). In graph A and B, the line represents moving averages (30 positions for total sequence in black; 10 positions for the separated nucleotides): blue for the first nucleotides of each codon in the Env reading frame (*env* N1), red for the second nucleotides (*env* N2) and green for the third nucleotides (*env* N3). The N1 variability has a major impact on Env and Rev and a small impact on Tat/Nef, the N2 variability has a major impact on Env and Tat/Nef and a small impact on Rev and finally the *env* N3 variability has a major impact on Tat/Nef and Rev and a small impact on Env. Above each plot are shown key regions of the different proteins. A: The first conserved region containing the endocytosis signal. B: The region containing the Kennedy-like sequences. C: The hydrophobic region. D: LLP-2 region. E: LLP-3 region. F: LLP-3 / LLP-1 interegion. G: LLP-1 region + position 166 for the stop codon.

**Figure 4:**
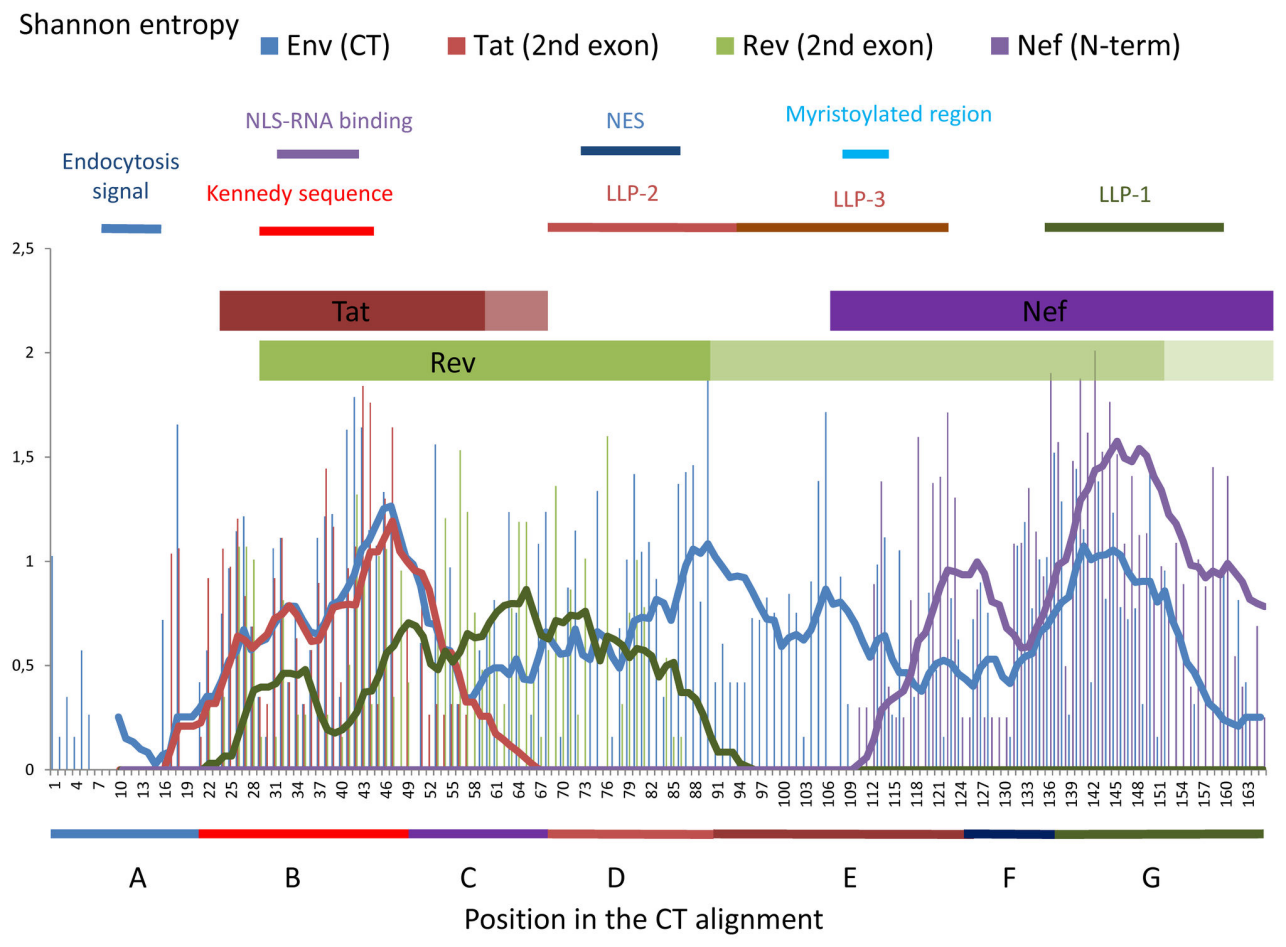
HIV-2 CT, Tat, Rev and Nef SE plot from our sequences. Above each plot are shown key regions of the different proteins. The *tat* 2^nd^ exon is expressed from the CT 16^th^ -17^th^ codon (16^th^ CT position was chosen in the plot for the first Tat position; in brown) until the CT 51^st^ - 52^nd^ codon. Some *tat* 2^nd^ exons continue to be expressed 6 codon later (shown in pale brown). The *rev* 2^nd^ exon is expressed from the CT 20^th^ -21^st^ codon (20^th^ CT position was chosen in the plot for the first Rev position) until the CT 104^th^ - 105^th^ codon (the last 14 Rev SE result are not shown due to the very complex situation created by insertion and deletion in this area; in green). The rev 2^nd^ exon of the group B is expressed until the CT 151^st^ -152^nd^ codon (in pale green) and some group A rev 2^nd^ exon continue to be expressed after the last CT codon (in faint green). *nef* is expressed from the CT 110^th^ -111^th^ codon (110^th^ CT position was chosen in the plot for the first Nef position) and continue to be expressed after the last CT codon. The different coloured lines represent the moving average of 10 positions. NLS-RNA binding: nuclear localisation signal of Rev binding to the genomic RNA, NES: Rev nuclear export signal, myristoylated region: refers to Nef. Above the plot, key regions of the different proteins are shown. A: The first conserved region containing the endocytosis signal. B: The region containing the Kennedy-like sequences. C: The hydrophobic region. D: LLP-2 region. E: LLP-3 region. F: LLP-3 / LLP-1 interegion. G: LLP-1 region + position 166 for the stop codon.

Figures 3a and 3b show the SE of the entire CT DNA/RNA coding region at the nucleotide level. In the first graph, we plotted all the SE for all 498 DNA/RNA positions (Figure 3a). To know which nucleotide(s) was the driver of the DNA/RNA variability, we plotted the 166 first nucleotides of the *env* codon (called *env* N1; *rev* N2; *tat*/*nef* N3), the 166 second nucleotides of the *env* codon (called *env* N2; *rev* N3; *tat*/*nef* N1) and 166 third nucleotides of the *env* codon (*env* N3; *rev* N1; *tat*/*nef* N2;) in a separate graph (Figure 3b). We then plotted the SE of each polypeptide sequence at their equivalent expressed positions in a single graph (Figure 4). Finally, we analysed region by region the impact of this high concentration of overlapping ORF. 

In the coding sequence of the conserved first 20 CT aa positions, the DNA/RNA region contained mainly the *env* ORF (region A, Table 4) and had low variability. The small nucleotide variability of this region was due to the variability of the *env* N3 that have a low impact on the Env protein aa variability (corresponding predominantly to synonymous substitutions). 

**Table 4:** Summary of the different SE analysed for each **CT** **DNA**/**RNA** coding regions.

Region (CT coding region nucleotide position to nucleotide position)	Average SE of the entire nucleotides	Average SE env N1	Average SE env N2	Average SE env N3
A (0 to 60)	0.222	0.162	0.166	0.340
B (61 to 141)	0.416	0.261	0.624	0.359
B group A*	0.131	0.088	0.202	0.102
B group B*	0.086	0.050	0.135	0.073
C (142 to 204)	0.356	0.227	0.301	0.539
C group A*	0.084	0.055	0.071	0.128
C group B*	0.098	0.058	0.033	0.202
D (205 to 273)	0.476	0.320	0.637	0.470
D/E (251 to 305)	0.658	0.638	0.601	0.734
E (274 to 375)	0.492	0.482	0.380	0.613
Nef (331 to 498)	0.412	0.424	0.244	0.570
Nef group A*	0.114	0.116	0.058	0.169
Nef group B*	0.104	0.114	0.062	0.134
F (376 to 418)	0.407	0.519	0.190	0.365
G (419 to 498)	0.454	0.433	0.286	0.686
G start (419 to 450)	0.670	0.632	0.503	0.885
G end (451 to 498)	0.288	0.241	0.081	0.539
CT (1 to 498)	0.414	0.345	0.389	0.510

A: coding region of the 20 first conserved positions. B: coding region of the Kennedy-like region. C: coding region of the hydrophobic region. D: coding region of the LLP-2. E: coding region of the LLP-3. F: coding region of the LLP-3/LLP-1 inter-region. G: coding region of the LLP-1. D/E: complex region of the end of the LLP-2 and the beginning of the LLP-3. Nef: region where the Nef protein is expressed. * Group A and B SE were normalized with the natural log of the number of sequences.

Within the Kennedy-like region, we observed a contrast between the variability of the nucleotides and that of the aa. The variability of the aa in the CT region was the highest, while the variability of the nucleotides was relatively lower and was close to the average SE of the entire DNA CT coding region (region B, Table 4). This was due to the fact that only the *env* N2 drove the variability in this region. Because the *env* N1 and N3 (respectively *rev* N2 and N1) were conserved, the Rev protein was relatively constant (Figure 4). Finally, the only variability of the *env* N2 leads to the co-variation of the Env and Tat proteins (Figure 4). 

The *tat* ORF ended five codons after the Kennedy-like coding region in most of our sequences, except for one sequence in which 6 codons were added to the consensus *tat* ORF (Genbank: KC748549). It is noteworthy that one *tat* ORF sequence was already finished at half the 2^nd^ exon due to a premature stop codon (Genbank: KC748550). In the sequences coding for the most hydrophobic CT region and the LLP-2 regions (regions C and D, respectively, Table 4), the *env* N1 were mainly conserved compared to the average SE for regions C and D. By contrast, the *env* N3 were the most variable in the coding region for the conserved CT hydrophobic region, and the *env* N2 were the most variable in the CT LLP-2 coding region. Thus, in the *env*/*rev* overlapping regions we observed that: the *env* N1 were always conserved, the *env* N2 were variable when the *env* N3 were conserved and the *env* N3 were variable when the *env* N2 were conserved. In conclusion the Rev and Env aa positions varied independently and mostly in an opposite way. By homology with the reference strain ROD, the regions where Rev was conserved contained the Rev nuclear localization signal (NLS), the RNA binding domain (RBD) and the nuclear export signal (NES) [23].

After those regions, the *rev* ORF ended a few positions after the beginning of the LLP-3 coding sequence in most of our group A sequences, but continued in all the group B sequences. The group B Rev continued to be expressed up to the codons of the CT position 151/152. In addition, in two group A sequences we did not find any *rev* stop codon in the *env* sequence. The SE value was high at the beginning of the LLP-3 region coding sequences, mainly because of deletions and insertions (named D/E, Table 4). When Nef started to be expressed, at the end of the LLP-3 coding region, the variability was mainly driven by the first and third nucleotides of the *env* codons (named Nef, Table 4). Thus, the nucleotides that created the high Nef variability (*env* N3) had no consequence on Env variability, and the nucleotides that created variability in Env (*env* N1) had no consequence on Nef variability. At the end of the LLP-1, where Env CT was the most constant, the DNA/RNA variability of the corresponding coding sequene seemed to be driven only by the *env* N3, allowing Nef variability and Env conservation (G end, Table 4). 

### Differences between the HIV-2 groups A and B

**Figure 5:**
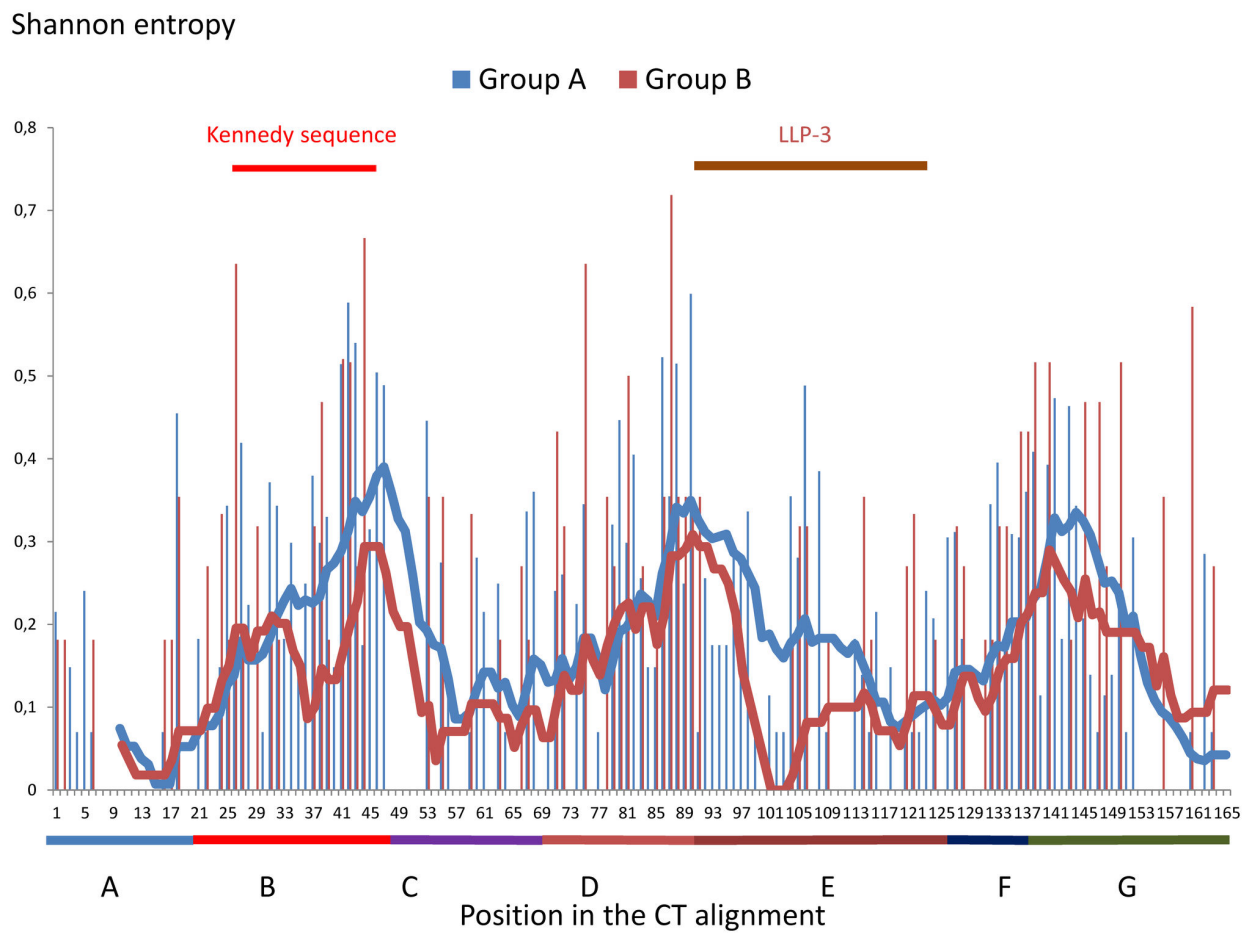
Normalised SE plot of the CT by HIV-2 group. 19 sequences from group A and 8 from group B are included. By contrast to the Figure 2, the SE are normalized by the natural log of the number of sequences to remove the entropy factor due to the sequences number. The lines represent the moving average of 10 positions (blue: group A, red: group B). The colours and letters below the plot represent the different studied regions: A: The first conserved region containing the endocytosis signal, B: The region containing the Kennedy-like sequences, C: The hydrophobic region, D: LLP-2 region, E: LLP-3 region, F: LLP-3 / LLP-1 inter-region, G: LLP-1 region + position 166 for the stop codon.

In the CT coding region, the SE (normalized by the natural log of the number of sequences) was similar between the HIV-2 groups A and B, except in the Kennedy-like region and at the beginning of the LLP-3 that was absent in sequences from group B. In the Kennedy-like region the mean SE was 50% higher in group A (Figure 5; region B group A and B Table 3), while the corresponding nucleotide sequence of the group A was more variable (region B group A and B, Table 4). This also means that the polypeptide expressed by the 2^nd^ exon of *tat* was more conserved in group B. We also observed differences in the group B *env* N3, which were significantly more variable than the group A *env* N3 in the coding region of the hydrophobic region (region C group A and B, Table 4). However, this difference did not lead to differences in CT variability. Finally, in most of the group A sequences the 2^nd^ exon of *rev* was two times smaller than its counterparts in group B creating an overlap with *nef*. This additional overlap had no consequence in the nucleotide variability (Nef group A and B, Table 4). 

## Discussion

We studied sequences of the HIV-2 Env C-terminal coding region from culture adapted strains and virus/provirus from clinical samples. We observed that stop codons were introduced prematurely in *env in vitro* but not *in vivo*. The position of the stop codon is interesting at two levels. First, it corresponds to a previously observed HIV-1 protease-cleavage site. In HIV-1, this site is important for resistance to the anti-fungus molecule Amphotericin B methyl ester (AME), which has an antiviral activity [24-26]. Furthermore, in the presence of AME, SIVmac selected the same stop codon than our HIV-2 strains [24]. But in the present study the selection of a premature stop codon happened without the presence of AME. Secondly the last aa of truncated Env is a proline, homologous to the first aa which anchors the HIV-1 Kennedy sequence in a model where the CT is partially outside the virus [1,14]. In our sequences, the region after this proline is very hydrophobic and mostly conserved. This suggests a potential membrane spanning region, and the presence of an external CT region similar to the HIV-1 Kennedy sequence. Some studies in HIV-1 showed the potential importance of the HIV-1 Env C-terminal part, corresponding to the HIV-2 Env C-terminal deleted part in cultured strains. This region was shown to play a role in the fusion process, the viral maturation, the packaging, and the anti-Env antibody recognition [27]. As the three first roles should act both *in vitro* and *in vivo*, the mandatory presence of the C-terminal region *in vivo*, suggests that immune recognition is the major selection pressure that favours a longer CT *in vivo* [40-42].

For practical reasons, it was not always possible to obtain a sequence either from viral RNA or proviral DNA in clinical samples. Global analysis could introduce a bias if both are very different. But our analysis detected the same variable and conserved regions in either RNA or DNA. The conserved regions are the endocytosis signal, the end of LLP-1 with the final di-leucine motif. The observed conservation of these regions is in line with their conservation among lentiviruses. The variable regions are the LLP-2, the Kennedy-like region and the beginning of the LLP-1. When compared to SIVmac, we found a supplementary conserved region known in SIVmac to be involved in NF-kB expression. However, that function is endorsed by another CT region in HIV-1, located in the LLP-2, which is not conserved between HIV types. It is not surprising to observe low HIV-1/HIV-2 conservation in the homologous LLP-2 and Kennedy sequences, as those two regions are already highly variable among HIV-1 groups and among our HIV-2 sequences [43]. Apart from being the least conserved regions, Kennedy-like region and LLP-2 region concentrated the mixtures of aa in population sequencing. This high variability can be explained because antibodies target those regions and they are therefore submitted to an evolutionary pressure [12–14,]. In contrast to the LLP-2, the Kennedy-like sequence is also variable *in vitro* when the sequences of cultured virus are compared with the reference sequences. This suggests that the Kennedy-like sequence is not exclusively variable *in vivo* while the LLP-2 is.

The HIV-2 DNA/RNA that encodes the variable Kennedy-like region encodes also the Rev conserved NLS and RBD. We showed that only the second nucleotides of *env* codons can vary to allow variability in Env with conservation in Rev (*env* N2= *rev* N3= *tat* N1). Consequently, the Kennedy-like region and the polypeptide expressed from the *tat* 2^nd^ exon co-evolve while Rev is conserved. Interestingly, one patient had a *tat* premature stop codon in the DNA/RNA Kennedy-like coding region (polypeptide from the 2^nd^ exon of *tat* shortened from 36 to 20 aa). This phenomenon does not seem to have an impact on virus replication since the patient had a high plasma viral load compared to the other patients (4^th^ highest viral load (Genbank:KC748550); Table 2). Furthermore, a *tat* premature stop codon is also present in HIV-1 strain BRU without changing the viral replication capacity [45].

Several sequences of this series showed insertions or deletions at the beginning of the putative LLP-3 region. As seven different cases were observed in this set, the beginning of the LLP-3 seems to be a complex region. Furthermore, we observed that the same patient could harbour virus or provirus with different insertions/deletions. This implies that this insertion/deletion phenomenon is a dynamic process in patients. We did not find a clear link between the patient’s viral load and the presence of such insertion or deletion. However 3 of the 4 highest viral load values were linked to sequences with the longest insertion: (Genbank:KC748550), (Genbank:KC748555) and ((Genbank:KC748564); Table 2). Compared to the consensus, all sequences from group B shared a same deletion in that area while this was not observed in sequences from group A. The beginning of the CT LLP-3 complex coding region corresponded to the C-terminal region of Rev. Both proteins are therefore affected by the insertions and deletions found in our DNA/RNA sequences. We also observed that some of the *rev* ORF continued beyond the consensus stop. This was the case for our entire group B sequences and two group A sequences: (Genbank: KC748548) and (Genbank:KC748578). In the case of group B the size of the *rev* second exon was doubled. The high variability of that supplementary Rev C-terminal region does not plead in favour of an important role. 

Finally, the end of the HIV-2 LLP-1 homologous region was highly conserved between our sequences and between primate lentiviruses. It was shown that this domain is implicated in Env incorporation within the envelope, HIV-1 infectivity, replication and fusion, and in neuronal cell death [37-39]. The high conservation between LLP-1 regions in primate lentiviruses implies that a similar effect could be found in SIV and HIV-2. 

## Conclusion

HIV-2 Env, Tat and Rev C-terminal regions are submitted to major length variations, either due to premature stop codons (only *in vitro* for Env and only *in vivo* for Tat), or to insertion and deletion inside the DNA/RNA sequences (for Env and Rev), or because of substitutions in the consensus stop codon (for Tat and Rev). The HIV-2 Env C-terminal part or CT contained three conserved and three variable regions. The conserved regions were the endocytosis signal and the regions homologous to the CT motif implied in SIVmac NF-kB expression and to the HIV-1 LLP-1 end. Two out of the 3 variable CT regions were homologous to HIV-1 Env regions harbouring epitope sequences: the Kennedy sequence and the lentiviral lytic peptide 2. Finally, this study highlighted that proteins expressed from the same nucleotide sequence in different reading frames mostly evolve independently as illustrated by Tat/Rev, Env/Rev and Env/Nef, but can also co-evolve as Tat and Env. 

## Materials and Methods

### Ethical approval

The project was approved by the CEBHF (Comission d’Ethique Biomédicale Hospitalo-Facultaire) of the Université Catholique de Louvain, Brussels, Belgium (2010/18OCT/322, B-40320109572). A written informed consent was obtained from patient.

### Strains and cell lines

The reference viruses ROD [46] and EHO [47] (obtained through the NIBSC, UK) were passaged several times on H9 cell lines (obtained through the NIH AIDS reagent reference program) [48-50]. The plasmid pKP59-ROD (used in [51]) was transfected on 293F cell lines (NIH AIDS reagent) [52]. The obtained virus was cultured several times on H9, MT2 and MT4 cell lines (NIH AIDS reagent) [53-57].

### Extraction of the clinical samples

Blood samples were taken on EDTA from HIV-2 infected patients. Viral RNA was extracted from the plasma with the Nuclisens extraction kit (Biomérieux, Marcy l'Etoile, France). Proviral DNA was extracted from whole blood with the Nucleospin blood DNA extraction Kit (Macherey-Nagel; Düren, Germany).

### Population Sequencing

We sequenced only the viral RNA for 17 of the patients, because the proviral DNA was not amplified for some or the samples were unavailable for the others. We sequenced both plasma RNA and provirus DNA in 4 of the patients. In 6 patients with low or undetectable viral load, only proviral DNA was sequenced. We developed a PCR amplification of the CT coding region with a nested PCR. Viral RNA or proviral DNA were amplified in a first reaction with the primers JR50 5’- CAGCAGGTTCTGCAATGGG -3’ and JR51 5’- CTCTCACTGTAATACATCCC -3’ at 300 nM, in a Master Mix with 2U of SuperScript-III enzyme (Life Technologies Ltd; Paisley, UK). The conditions used were 60 min at 45°C when the template was RNA, followed by 2 min at 94°C and 40 cycles of 30 sec at 94°C, 45 sec at 56°C, 2 min 30 à 72°C. A nested PCR using primers JR52 5’- TGGCCGGGATAGTGCAGC -3’ and JR53 5’- AACATCCCTTCCAGTCCC -3 at 300 nM was then performed using 2uL of the first PCR product with KOD enzyme master mix (Merck Millipore; Darmstadt, Germany). A touch-down protocol was used as follows: 2 min at 95°C and 10 cycles of 20 sec at 95°C, 10 sec 65°C to 56°C pendant (1°C less every cycle), 30 sec 70°C following by 30 cycles of 20 sec at 95°C, 10 sec 56°C, 30 sec 70°C.

The nested PCR products were purified with the QIAquick PCR purification kit (Qiagen; Venlo, Netherlands) and were sequenced with the primers JR49: 5’-GGTTTGACTTAACCTCCTGG-3’, JR52, JR53, JR54: 5’-GACAACAAGAACTGTTGCG-3’ and JR55: 5’-TGTCATTGGYCTYAGTGG-3’. We used the BigDye Terminator v3.1 (Applied Biosystems; Foster City, USA) for the sequencing reaction. The product was purified with the BigDye XTerminator Purification Kit (Applied Biosystems; Foster City, USA) and run in a capillary electrophoresis on the ABI3130 sequencing platform (Applied Biosystems; Foster City, USA). 

### Availability of supporting data

The data set supporting the results of this article are available in the Genbank repository. The accession numbers are from (Genbank:KC748535) to (Genbank:KC748580). For the “Analysis of the sequences”, “Influence of Tat, Rev and Nef on HIV-2 CT variability” and “Differences between the HIV-2 groups A and B” sections accession numbers of the sequences used can be found in Figure 1. For the “HIV-2 CT sequence variability over time in infected individuals” section accession numbers of sequences used were (by alignment):   [(Genbank:KC748536) (Genbank:KC748537)], [(Genbank: KC748538)  (Genbank:KC748539)  (Genbank:KC748540)  (Genbank:KC748541) (Genbank:KC748542) (Genbank:KC748543)], [(Genbank:KC748572) (Genbank:KC748573)], [(Genbank:KC748575) (Genbank:KC748576) (Genbank:KC748577)],[(Genbank:KC748545) (Genbank:KC748546)],[(Genbank:KC748567) (Genbank:KC748568)],[(Genbank:KC748553) (Genbank:KC748554)], [(Genbank:KC748569) (Genbank:KC748570) (Genbank:KC748571)] and [(Genbank:KC748560) (Genbank:KC748561)] For the “Provirus-virus sequences comparison” section accession numbers of the provirus sequences used in the alignment were  (*Genbank:KC748565*), (Genbank:KC748566), (*Genbank:KC748568*), (Genbank:KC748569), (*Genbank:KC748573*)*,* (*Genbank:KC748574*), (Genbank:KC748575), , (Genbank:KC748578), (Genbank:KC748579) and (Genbank:KC748580) and accession numbers of the virus sequences used In the alignment were (Genbank:KC748535), (*Genbank:KC748536*), (Genbank:KC748538), (Genbank:KC748544), (*Genbank:KC748545*), (*Genbank:KC748547*), (*Genbank:KC748548*), (Genbank:KC748549), (Genbank:KC748550), (Genbank:KC748551) (Genbank:KC748552), (Genbank:KC748553), (Genbank:KC748555), (Genbank:KC748556), (Genbank:KC748557), (Genbank:KC748558), (Genbank:KC748559), (Genbank:KC748560), (Genbank:KC748562), (Genbank:KC748563) and (Genbank:KC748564) (The numbers in italics represent the accession numbers of the sequences used for the direct provirus virus comparison). 

### Sequence analysis and alignment

Sequences were assembled and aligned using the IDNS database HIV-2 module (Smartgene; Zug, Switzerland). The program used for the multiple alignments was Multalin [58]. Shannon entropy was calculated by the program Entropy one [59]. The nucleotide sequences were translated by Transeq [60]. Finally, the Kyte Doolittle measure was performed in the Expasy portal [61].
